# α-Amyrin and β-Amyrin Isolated from *Celastrus hindsii* Leaves and Their Antioxidant, Anti-Xanthine Oxidase, and Anti-Tyrosinase Potentials

**DOI:** 10.3390/molecules26237248

**Published:** 2021-11-29

**Authors:** Tran Duc Viet, Tran Dang Xuan, La Hoang Anh

**Affiliations:** Graduate School for International Development and Cooperation, Hiroshima University, Higashi-Hiroshima 739-8529, Japan; viettran1609@gmail.com (T.D.V.); hoanganh6920@gmail.com (L.H.A.)

**Keywords:** α-amyrin, β-amyrin, GC-MS, ESI-MS, NMR, chromatography, antioxidant, anti-xanthine oxidase, anti-tyrosinase

## Abstract

*Celastrus hindsii* is a popular medicinal plant in Vietnam and Southeast Asian countries as well as in South America. In this study, an amount of 12.05 g of an α-amyrin and β-amyrin mixture was isolated from *C. hindsii* (10.75 g/kg dry weight) by column chromatography applying different solvent systems to obtain maximum efficiency. α-Amyrin and β-amyrin were then confirmed by gas chromatography-mass spectrometry (GC-MS), electrospray ionization-mass spectrometry (ESI-MS), and nuclear magnetic resonance (NMR). The antioxidant activities of the α-amyrin and β-amyrin mixture were determined via 2,2-diphenyl-1-picrylhydrazyl (DPPH) and 2,20-azinobis (3-ethylbenzothiazoline-6-sulfonic acid) (ABTS) assays with IC_50_ of 125.55 and 155.28 µg/mL, respectively. The mixture exhibited a high potential for preventing gout by inhibiting a relevant key enzyme, xanthine oxidase (XO) (IC_50_ = 258.22 µg/mL). Additionally, an important enzyme in skin hyperpigmentation, tyrosinase, was suppressed by the α-amyrin and β-amyrin mixture (IC_50_ = 178.85 µg/mL). This study showed that *C. hindsii* is an abundant source for the isolation of α-amyrin and β-amyrin. Furthermore, this was the first study indicating that α-amyrin and β-amyrin mixture are promising in future therapies for gout and skin hyperpigmentation.

## 1. Introduction

*Celastrus hindsii* Benth. belongs to the Celastraceae family, which is found in many countries such as Laos, Thailand, Vietnam, and South America [1]. In Vietnam, since ancient times, *C. hindsii* has been used as a traditional medicine to treat numerous diseases such as cancer, ulcers, and inflammation [2]. Previous studies have shown that *C. hindsii* contains many precious compounds such as rosmarinic acid [1], terpenoids [3], alkaloids [4], phenolics, and flavonoids [2]. However, the use of this plant as folk medicine was mainly based on human experience over its long history. Its therapeutic mechanism has not been verified, and the major compounds and their actual contribution to the biological properties of *C. hindsii* have not been clearly investigated.

α-Amyrin and β-amyrin are widely known as a natural mixture of triterpenes. Their pharmacological potentials have been reported in previous studies [5], including antitumor [6], anti-inflammatory [7], anxiolytic [8], and hepatoprotective [9] effects. α-Amyrin and β-amyrin can be found in numerous plant species such as *Solanum lycopersicum*, *Liriodendron tulipifera*, *Olea europaea*, *Aesculus hippocastanum*, *Aloe vera*, *Betula alba*, *Calendula officinalis*, *Coffea arabica*, *Malus domestica*, *Viscum album*, *Syzygium aromaticum*, *Pisum sativum*, *Brassica oleracea*, *Protium kleinii*, *Symplocos cochinchinensis*, *Alstonia boonei*, *Swertia longifolia*, *Canarium tramdenum*, *Byrsonima crassifolia*, etc. [10,11]. However, the isolation and accurate identification as well as quantification of these compounds in plant sources have been limited. The separation of these compounds for the purpose of treating diseases has not been specifically proven. On the other hand, *C. hindsii* has been used as a medicinal plant for a long time [1,2,3]. The distribution area of this plant is also wide, contributing an abundant source of raw materials [2]. Additionally, we have found that α-amyrin and β-amyrin are the dominant compounds in *C. hindsii*. Therefore, the isolation and purification of α-amyrin and β-amyrin from *C. hindsii* are significantly needed. In the current research, we aimed to obtain the maximum amount of these two compounds from this species. In our study, column chromatography supported by gas chromatography-mass spectrometry (GC-MS), electrospray ionization mass spectrometry (ESI-MS), and nuclear magnetic resonance (NMR) were used to isolate and purify α-amyrin and β-amyrin from *C. hindsii*. In addition, the potentials of α-amyrin and β-amyrin for antioxidant, antigout, and anti-skin hyperpigmentation were investigated for the first time via in vitro assays.

In humans, oxidative stress is an important physiological process involved in many chronic diseases such as diabetes and cancer. Some studies have indicated the existences and contributions of oxidative stress in several disorders via inflammation [12]. Oxidative stress can induce an inflammatory process and vice versa in chronic disorders [13]. Therefore, screening the antioxidant capacities of target compounds was the first important step of our tests for biological activities. Among chronic diseases, gout is a serious disorder caused by increasing uric acid in the blood. This disease seriously affects the populations of many countries in the world [14]. In terms of the underlying mechanism, xanthine oxidase (XO) plays an important role in the elevation of uric acid in the blood, leading to hyperuricemia, followed by the development of gout [15]. Until now, only allopurinol and febuxostat have been acknowledged for their role in inhibiting XO in the treatment of hyperuricemia. However, they also have many undesirable effects such as hypersensitivity syndrome, hepatitis nephropathy, eosinophilia, fever, vasculitis, and skin rash; therefore, further research into XO inhibition compounds is necessary [16,17]. Additionally, other potential compounds inhibiting the activity of xanthine oxidase could be promising source in the development of novel drugs for gout.

Another chronic syndrome, as skin hyperpigmentation, causes changes in skin color, loss of aesthetics, and an increase in the risk of skin cancer [18]. In the formation of skin hyperpigmentation, tyrosinase is a key regulatory enzyme in the proliferation of browning pigments. It plays an important role in controlling the amount of melanin, which is crucial to the health of the skin [19]. Thus, tyrosinase inhibitors are often used in the development of medicines and cosmetics. Furthermore, the synergic inhibition of oxidative stress, xanthine oxidase, and tyrosinase could be a potential approach to the generation of alternative drugs, which can prevent multiple human health problems [20].

For this purpose, many natural and semisynthetic inhibitors have been developed by various methods. The aforementioned rationales prompted us to conduct research focusing on the isolation of α-amyrin and β-amyrin from *C. hindsii* and examine their antioxidant, anti-xanthine oxidase, and anti-tyrosinase activities. This study contributes to the development of natural-based products for preventing chronic disorders including gout and skin hyperpigmentation.

## 2. Results

### 2.1. Isolation of α-Amyrin and β-Amyrin from Leaves of C. hindsii

A dried powder of *C. hindsii* (1.12 kg) was extracted by methanol to collect crude extract (260.80 g). Then, it was partitioned with three solvents, including water, hexane, and ethyl acetate. Next, a preliminary activity assessment and thin-layer chromatography were conducted. The results showed that the ethyl acetate mixture had the most potential, and it was used for column chromatography separation. After checking by thin-layer chromatography, the ethyl acetate extract was the most active for the target compounds of α-amyrin and β-amyrin. This extract was then used to run column chromatography. The results showed that nine fractions (C1–C9) contained the α-amyrin and β-amyrin. These fractions were combined to run a second-time column chromatography. Finally, 12.05 g of an α- and β-amyrin mixture was obtained.

### 2.2. Identification of α-Amyrin and β-Amyrin by GC-MS and ESI-MS

**Figure 1:**
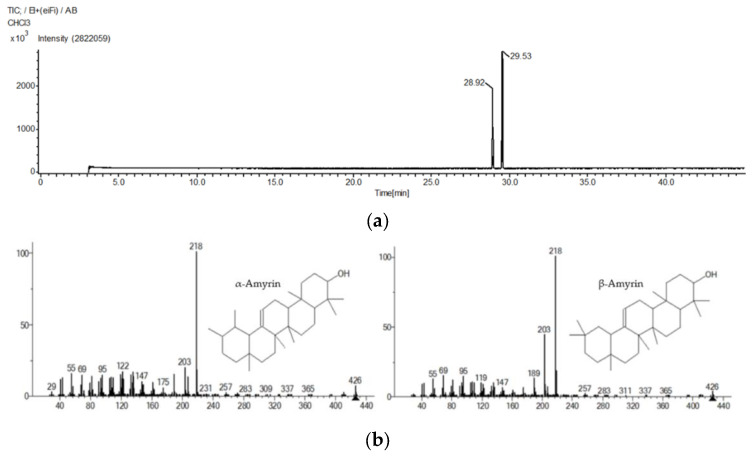
GC-MS chromatogram (**a**) and mass spectra (**b**) of α-amyrin and β-amyrin.

Based on the GC-MS results (Figure 1), the presence of α-amyrin and β-amyrin was confirmed. The retention times of α-amyrin and β-amyrin were 29.53 and 28.92 min, respectively (Table 1).

**Figure 2:**
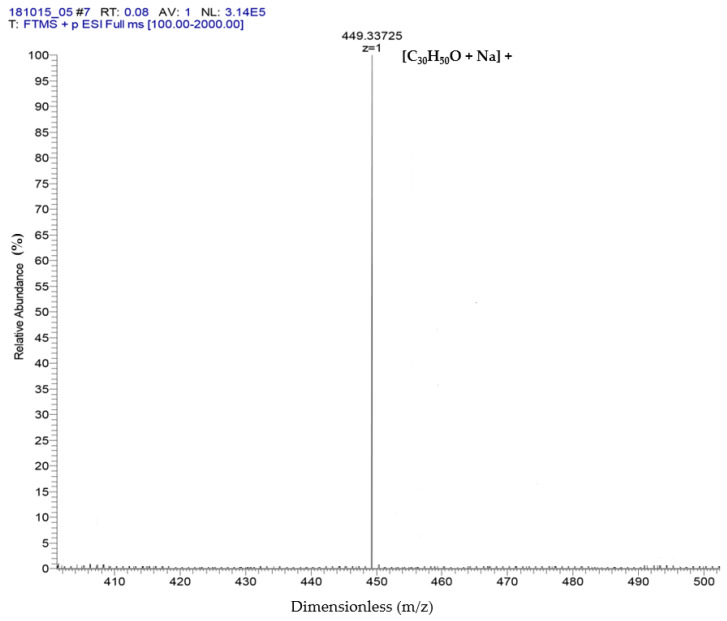
Identification of α-amyrin and β-amyrin by ESI-MS.

The results of ESI-MS showed the molecular weight and formula of α-amyrin and β-amyrin, which were verified as dimensionless at 449.33 + 1 *m*/*z* and formula [C_30_H_50_O + Na]^+^ (Figure 2). These results referred to the studies of Banerjee et al. [21] and Pinto et al. [22] to support the results of the GC-MS in which two compounds were isolated: α-amyrin and β-amyrin.

### 2.3. Elucidation of α-Amyrin and β-Amyrin by NMR

**Figure 3:**
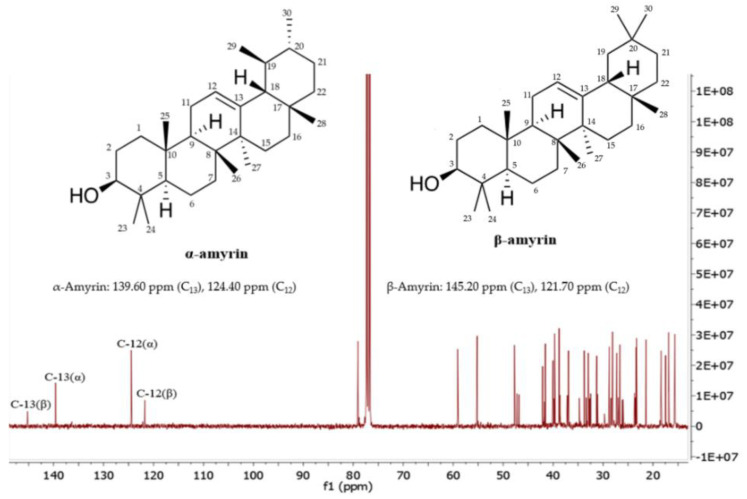
^13^C-NMR spectrum of the mixture of α-amyrin and β-amyrin measured in CDCl_3_ (125 MHz).

Figure 3 shows the ^13^C NMR spectrum of the mixture of α-amyrin and β-amyrin. The signals at 139.60 ppm and 145.20 corresponded to C_13_, whereas the signals at 124.40 and 121.70 ppm corresponded to C_12_ of α-amyrin and β-amyrin, respectively. The other signals were assigned as shown in (Table 2), which are in agreement with the published literature [23,24].

**Figure 4:**
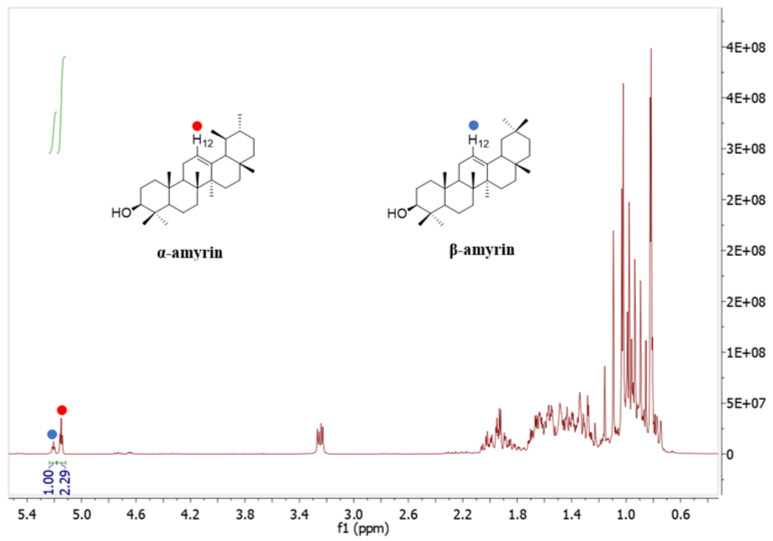
^1^H-NMR spectrum of the mixture of α-amyrin and β-amyrin measured in CDCl_3_ (400 MHz).

The ^1^H NMR spectrum (Figure 4) illustrates that the two signals at 5.18 ppm (H-12, 1H) and 5.13 ppm (H-12, 1H) were assigned to β-amyrin and α-amyrin, respectively. The ratio of α-amyrin and β-amyrin was (2.29:1), which was calculated based on the integration value of these two signals [23,24].

**Figure 5:**
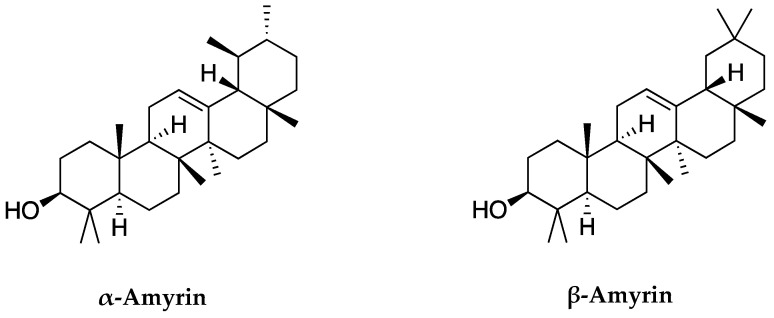
Chemical structures of α-amyrin and β-amyrin identified in *C. hindsii*.

Based on the chemical structures of α-amyrin and β-amyrin in Figure 5, these two compounds share the same molecular weight and chemical formula. The difference only comes from the methyl group (CH_3_) at C_30_ and C_29_ carbon positions. In α-amyrin structure, the methyl groups of C_29_ and C_30_ are attached at two separate positions. One is directed forward and one to back. While in β-amyrin, the methyl groups of C_29_ and C_30_ are attached to the same position and are directed forward. This is also the basis for distinguishing the two substances as well as identifying these two compounds.

### 2.4. Quantitative Analysis of α-Amyrin and β-Amyrin in C. hindsii Leaves

A total of 12.05 g of α-amyrin and β-amyrin mixture was isolated from 1.12 kg of *C. hindsii* dried leaves. The ratio of α-amyrin and β-amyrin in the mixture was determined as (2.29:1) based on the NMR results (Figure 4). The content of α-amyrin and β-amyrin mixture in leaves of *C. hindsii* was quantified by the method of Shantos et al. [24] as 10.75 g/kg dry weight (DW), and the ratio of α-amyrin and β-amyrin in the mixture was 69.60% and 30.40%, respectively, equivalent to 7.48 g/kg DW of α-amyrin and 3.27 g/kg DW of β-amyrin (Table 3).

### 2.5. Antioxidant, Anti-Xanthine Oxidase, and Anti-Tyrosinase of α-Amyrin and β-Amyrin from C. hindsii

The antioxidant activities of α-amyrin and β-amyrin were determined via DPPH and ABTS free-radical scavenging assays. BHT was used as a standard inhibitor. The results were summarized in Table 4. The IC_50_ values of α-amyrin and β-amyrin were 125.55 and 155.28 µg/mL for DPPH and ABTS assays, respectively. For XO inhibitory potential, α-amyrin and β-amyrin mixture exhibited strong activity, with an IC_50_ value of 258.22 µg/mL. For suppression of tyrosinase, the mixture had an IC_50_ value of 178.85 µg/mL. Allopurinol and kojic acid were used as standard inhibitors for XO (IC_50_ = 7.58 µg /mL) and tyrosinase (IC_50_ = 15.55 µg /mL), respectively.

## 3. Discussion

*Celastrus hindsii* has shown numerous biological activities in previous research including preventing inflammation, antitumor effects, inhibiting HIV replication, etc. [25,26]. *C. hindsii* has been reported to contain many valuable chemical components, including sesquiterpenes, triterpenes, alkaloids, and flavonoids [27,28]. *C. hindsii* is now attracting greater international research attention [29]. In this study, *C. hindsii* was used to isolate and purify α-amyrin and β-amyrin. These compounds are classified as triterpenes for which anti-obesity [30], anxiolytic [31], antidepressant and antiplatelet [32], antinociceptive [33], liver-protective [34], gastroprotective [35], anti-inflammatory [36], antiulcer, antihyperlipidemic, and antitumor [37] properties have been reported. However, the potential for these compounds to inhibit gout and skin hyperpigmentation was investigated for the first time in this study.

For the isolation and purification of α-amyrin and β-amyrin, column chromatography was used in this study with different extracting solvents. This is a popular technique, which is widely used to isolate individual substances of interest in mixtures from large to small sizes and can applied both at the laboratory scale and in industrial production [38]. To support the isolation and purification, GC-MS, ESI-MS, and NMR were performed. Gas chromatography-mass spectrometry (GC-MS) is an analytical method that involves the combination of gas chromatography and mass spectrometry to identify different components within a test sample [39]. In addition, this technology allows the identification of trace elements, even in tiny amounts, and composition of compounds present in an entity after it has been decomposed [40]. This technology allows both qualitative and quantitative conversions to the analytical mode in the form of ions [41]. Electrospray ionization mass spectrometry (ESI-MS) was developed as a significant technique to provide the sensitive, robust, and reliable tool for research, conducted at atmospheric pressure and at a moderate temperature, using non-volatile and thermally labile biomolecules that are not amenable to analysis by other conventional techniques [42]. Nuclear magnetic resonance (NMR) is a technology used to determine the structure of molecules, both crystalline and noncrystalline based on results from the specific properties of certain atomic nuclei of hydrogen and carbon. ^1^H spin 1/2 occurs in many biological systems. The NMR proton system produces the displacement of hydrogen in a narrow range with a sharp, signal thereby giving fast quantum results in a short time [43]. The ^13^C spin 1/2 method has been used widely in NMR conduction. Although it occurs at a small proportion in nature (approximately 1.1%), it is associated with nuclear fission. Therefore, the sensitivity is low, the output signal is sharp, the effects of spin are prevented, and the mass spectrometry is not disturbed [44]. As a result, a total of 12.05 g of α-amyrin and β-amyrin were isolated and purified from *C. hindsii* leaves and were able to be confirmed based on the GC-MS, ESI-MS, and NMR results.

In other studies, α-amyrin and β-amyrin have been found in peas (*Pisum sativum*) [45] and cabbages (*Brassica oleracea*) [46]. Especially, an α-amyrin and β-amyrin mixture (2.40 g/kg DW) was isolated from *Protium kleinii* [32]. β-Amyrin was determined in *Symplocos cochinchinensis* [47], *Alstonia boonei*, and *Swertia longifolia* at the amounts of 1.70; 8.00; and 2.00 g/kg DW, respectively [48]. In the case of α-amyrin, the compound was quantified in *Melastoma malabathricum* [49] and *Swertia longifolia* [50], in which the contents were 0.60 and 1.00 g/kg, respectively. In *Canarium tramdenum* and *Byrsonima crassifolia,* α-amyrin and β-amyrin mixtures were determined at 1.52 and 9.00 g/kg DW [51,52]. α-Amyrin was studied in plant cell cultures of *Centella asiatica* [53]. In this study, a total of 12.05 g of α-amyrin and β-amyrin mixture was obtained from 1.12 kg of *C. hindsii* dried leaves, equivalent to 10.75 g/kg DW (Table 3). The contents of α-amyrin and β-amyrin were determined based on NMR results (Table 2, Figure 3 and Figure 4). α-Amyrin and β-amyrin accounted for 7.48 and 3.27 g/kg DW, respectively (Table 3), which are significantly higher than those found in other plant species. This demonstrated that *C. hindsii* is a potential source to isolate α-amyrin and β-amyrin. A further step in our study was to evaluate the antioxidant, anti-xanthine oxidase, and anti-tyrosinase properties of α-amyrin and β-amyrin, which is discussed as follows.

Oxidative stress is an imbalance between reactive oxygen/nitrogen species (ROS/RNS), leading to the loss of antioxidant capacity. Oxidative stress has been linked to many diseases such as aging, cardiovascular disease, and cancer [54]. Free-radical-induced damage in oxidative stress has been confirmed as a contributor to the pathogenesis of many chronic health problems such as neurodegenerative, emphysema, cardiovascular and inflammatory diseases, cataracts, and cancer [55]. Oxidative stress is implicated in many diseases [56]. An irreversible progression of oxidative decay caused by reactive oxygen species also exerts its negative influence on the biology of aging, leading to the impairment of physiological functions, promoting disease incidence, and reducing life span [57]. α-Amyrin and β-amyrin isolated from *Myrcianthes pungens* leaves were reported as having antioxidant potential [58]. The antioxidant agent of α-amyrin and β-amyrin has been shown to combat acute pancreatitis [59]. Oxidative stress is closely related to inflammatory diseases [12,13]. Many studies have shown the strong anti-inflammatory ability of α and β-amyrins [5,45]. Thus, this assessment of α- and β-amyrin’s antioxidant abilities was consistent with previous findings. Both *C. hindsii* α amyrin and β-amyrin showed antioxidant capacity [2,51]. The results of this evaluation of α- and β-amyrin’s antioxidant potential by two methods, DPPH and ABTS (Table 4), are important additional evidence of the value of α and β-amyrin and *C. hindsii* in term of antioxidant capacity.

Gout is a chronic disease caused by the deposition of monosodium urate crystals [60]. The changes of urate transport in the kidneys and gut play an important role in elevation of uric acid in the blood, which is the cause of gout [61]. Xanthine oxidase is an enzyme that converts hypoxanthine into uric acid, and the strong activity of this enzyme leads to increased uric acid concentration, causing the deposition of monosodium urate crystals that cause gout [62]. This syndrome is closely related to inflammatory reactions; therefore, gout often manifests as monoarthritic of the extremities, so anti-inflammatory agents are widely used to treat gout [63,64]. This was the basis for the evaluation of the anti-gout ability of α and β-amyrin as they are anti-inflammatory compounds [65]. The results of α- and β-amyrin’s anti-xanthine oxidase potential were shown in (Table 4). The value of this mixture was confirmed. In addition, this evaluation further emphasized the utility of these compounds in the treatment of gout.

Tyrosinase is an enzyme that catalyzes the synthesis of melanin [5]. Tyrosinase has been studied extensively in the treatment of malignant cancers [66]. Anti-tyrosinase has been studied in antibodies in melanoma and vitiligo [67]. Tyrosinases are exploited for a variety of biotechnological and environmental applications. Their potential has been applied in food, pharmaceutical, and environmental sectors [68]. Tyrosinase is involved in neurodegenerative disorders, such as Parkinson’s disease, and in melanin-browning reactions important to the cosmetics and food industries [69]. Tyrosinase attracted the interest of immunologists because it was known that melanoma patients could form an immune response to antigens related to melanogenesis [70]. This is the first study of α-amyrin and β-amyrin to consider anti-tyrosinase potential, and our results support the development of α-amyrin and β-amyrin as therapeutics for skin hyperpigmentation.

## 4. Materials and Methods

### 4.1. Materials

The plant materials of *C. hindsii* were collected in Cao Duong commune, Luong Son district, Hoa Binh province, Vietnam. Fifty mature plants were randomly selected for leaf harvest. The collected *C. hindsii* plants were in the age range of over 5 years. They have grown naturally. *C. hindsii* plants were healthy and free from pests and diseases, without the use of fertilizers, pesticides, and stimulants. The materials were authenticated and sterilized. Specimens (No. PPBC170506) were preserved before further experiments were caried out. The study was conducted from March to December in 2020.

### 4.2. Preparation of Plant Extract

A total of 1.12 kg of *C. hindsii* dried leaves was used for the experiment. The material was redried for 2 days at 40 °C. After that, the obtained sample was soaked in 10 L of MeOH for 30 days at room temperature. It was then filtered and concentrated by a rotary evaporator (SB-350-EYELA, Tokyo Rikakikai Co., Ltd., Tokyo, Japan) to obtain 260.80 g of crude extract. This crude extract was separated by three solvents (water, hexane, and ethyl acetate).

### 4.3. Fractionation of Ethyl Acetate Extract

The ethyl acetate extract showed the strongest inhibition by thin-layer chromatography test, was subjected to a normal phase of column chromatography (20 mm diameter × 500 mm height, Climbing G2, Tokyo, Japan) over silica gel (size A 60, 200–400 mesh particle size) (Sigma Aldrich, Tokyo, Japan). This process yielded 9 fractions with following eluents: C1 in CHCl_3_, C2 in CHCl_3_:MeOH (99:1) flask 1–20, C3 in CHCl_3_:MeOH (99:1) flask 21–40, C4 in CHCl_3_:MeOH (99:1) flask 41–60, C5 in CHCl_3_:MeOH (99:1) flask 61–80, C6 in CHCl_3_:MeOH (99:1) flask 81–100, C7 in CHCl_3_:MeOH (97:3), C8 in CHCl_3_:MeOH (95:5), and C9 in CHCl_3_:MeOH (9:1). Then, these fractions were combined to run column chromatography for the second time.

### 4.4. Identification α-Amyrin and β-Amyrin by Gas Chromatography-Mass Spectrometry (GC-MS)

The composition of substances in *C. hindsii* was determined by the GC-MS system. An aliquot of 1 µL sample dissolved in chloroform was added to the GC-MS system (JMS-T100 GCV, JEOL Ltd., Tokyo, Japan). The column was DB-5MS, 30 mm length, 0.25 mm internal diameter, and 0.25 µm in thickness (Agilent Technologies, JW Scientific Products, Folsom, CA, USA). Helium was used as carrier gas, and ratio was 5:1. The method to operate the GC oven temperatures was conducted, the temperature was 50 °C, and program rate was 10 °C up to 300 °C with holding time of 20 min. The injector and detector temperatures were 300–320 °C, respectively. The mass range scanned from 28 to 800 amu. The control of GC-MS system and data processing were identified following JEOL’s GC-MS Mass Center System Version 2.55a (JEOL Ltd., Tokyo, Japan). The peak areas of compounds (>3%) analysis were identified as principal substances [71].

### 4.5. Electrospray Ionization-Mass Spectrometry (ESI-MS) Analysis

ESI-MS analysis was carried out in negative and positive ion mode. The capillary temperature was conducted at 140 °C (12 °C for S2), and the spray voltage was 3.0 KV (2.7 KV for S2). The measurements were calculated in the positive mode with an ion spray voltage of 3000 V and capillary temperature of 350 °C. Data peaks were estimated within the range of 280–1000 *m*/*z* [21].

### 4.6. Nuclear Magnetic Resonance (NMR) Data of α-Amyrin and β-Amyrin

α-Amyrin: ^1^H NMR (400 MHz, CDCl_3_): 3.24 (dd, J = 10.9 and 5.1 HzH-3), 5.12 (t, J = 3.6 HzH-12). δ ^13^C NMR (125 MHz, CDCl_3_): 38.8 (C-1), 27.3 (C-2), 79.1 (C-3), 38.8 (C-4), 55.2 (C-5), 18.4 (C-6), 32.9 (C-7), 40.0 (C-8), 47.7 (C-9), 36.9 (C-10) 23.3 (C-11), 124.4 (C-12), 139.6 (C-13), 41.5 (C-14), 28.1 (C-15), 26.6 (C-16), 33.8 (C-17), 59.1 (C-18), 39.7 (C-19), 39.6 (C-20), 31.3 (C-21), 40.0 (C-22), 28.1 (C-23), 15.7 (C-24), 15.6 (C-25), 17.4 (C-26), 23.4 (C-27), 28.8 (C-28), 16.9 (C-29), 21.4 (C-30) [23,24].

β-amyrin: ^1^H NMR (400 MHz, CDCl_3_): 3.24 (dd, J = 10.9 and 5.1 Hz, H-3), 5.18 (t, J = 3.6 Hz, H-12). δ ^13^C NMR (125 MHZ, CDCl_3_): 38.6 (C-1), 27.2 (C-2), 79.0 (C-3), 39.8 (C-4), 55.2 (C-5), 18.4 (C-6), 32.5 (C-7), 41.7 (C-8), 47.6 (C-9) 37.0 (C-10), 23.7 (C-11), 121.7 (C-12) 145.2 (C-13), 42.1 (C-14), 26.9 (C-15), 26.2 (C-16), 32.7 (C-17), 47.2 (C-18), 46.8 (C-19), 31.1 (C-20), 34.7 (C-21), 37.1 (C-22), 28.1 (C-23), 15.6 (C-24), 15.5 (C-25), 16.8 (C-26), 26.0 (C-27), 28.4 (C-28), 33.3 (C-29), 23.5 (C-30) [23,24].

### 4.7. Xanthine Oxidase (XO) Inhibitory Activity

The XO activity was assessed spectrophotometrically, which was based on the research of Andriana et al. [72] with some modifications. In brief, a mixture of 20 µL of sample and 40 µL of 70 mM phosphate buffer (pH = 7.5) and 40 µL of enzyme solution (0.01 units/mL in buffer) was incubated for 8 min at room temperature. After that, 60 µL of xanthine substrate (300 µM in buffer) was injected. The mixture was incubated at room temperature. The reaction was stopped by adding 20 µL of HCl 1M. The result was read at 290 nm by a microplate reader (Multiskan^TM^ microplate spectrophotometer, Fisher Scientific, Osaka, Japan). XO inhibitory activity was used according to formula below:

(1) $$\%\;\mathrm{Inhibition}=\left\{\frac{\left(E-C\right)-\left(T-W\right)}{E-C}\right\}\times 100$$

where E is the activity of the enzyme without test sample. C is the control of experiment without test sample.

T is the activity of test sample. W is the blank (without test). Allopurinol (10–100 µg/mL) was used as a positive control. IC_50_ values were calculated from the mean values of the percentage inhibition data.

### 4.8. 2,2-Diphenyl-1-picrylhydrazyl (DPPH) Free Radical Scavenging Activity

The antioxidant activity of the α-amyrin and β-amyrin mixture was estimated via DPPH free radical scavenging method described by Quy et al. [73]. In details, a mixture containing 50 µL of α-amyrin and β-amyrin with 0.25 µL of 0.5 mM DPPH solution and 0.5 µL of 0.1 M acetate buffer (pH 5.5) was prepared and placed in the dark for 30 min under ambient conditions. The absorbance of the reaction was recorded at 517 nm using a microplate reader (Multiskan^TM^ Microplate Spectrophotometer, Thermo Fisher Scientific, Osaka, Japan). Methanol and BHT (10–50 ppm) were used as negative and positive controls, respectively. The antioxidant capacity of the tested samples was calculated using the following equation:

DPPH radical scavenging activity (%) = [(C − S)/C]/100(2)

where S is the absorbance of sample. C is the absorbance of the reaction without the sample. The result was expressed as IC_50_ value, which determined the concentration of the sample required to scavenge 50% of DPPH.

### 4.9. 2,20-Azinobis (3-Ethylbenzothiazoline-6-sulfonic Acid) (ABTS) Cation Scavenging Activity

The ABTS method was used to evaluate the antioxidant capacity of *C. hindsii* [73]. The ABTS solution was created by adding 7 mM ABTS to 2.45 mM potassium persulfate solution. After that, the mixture was incubated in the dark at room temperature for 16 h.

MeOH was pushed to achieve an absorbance of 0.68 ± 0.05 at 734 nm. A volume of 24 µL of each sample was added to 120 µL of ABTS solution, and then the mixture was incubated in the dark under ambient condition for 30 min. The absorbance was measured at 734 nm by a microplate reader. BHT (5–125 µg/mL) was considered as a standard inhibitor, and MeOH was a negative control. The ABTS radical scavenging activity was evaluated with the same method as presented for DPPH assay.

### 4.10. Tyrosinase Inhibition

The prepared components included 20 mM potassium phosphate buffer (pH = 6.8) and tyrosinase solution (500 units/mL in buffer). The substrate solution was L-tyrosine (2 mM in distilled water). The sample was dissolved in DMSO. For the reaction, the combination of 20 µL of test sample, 20 µL of enzyme, and 120 µL of buffer was incubated for 5 min at 25 °C. The final solution’s absorbance was calculated at 470 nm. Kojic acid was used as a standard inhibitor, and DMSO was used as a negative control. The inhibition potential was evaluated by the following formula:

% Inhibition = (C − S)/C × 100(3)

C: Absorbance of the reaction with DMSO after the substrate control’s blank; S: Absorbance of the reaction with sample (without enzymatic reaction on substrate). IC_50_ results of the sample against tyrosinase were calculated with the same method of antiradical assays [20].

### 4.11. Statistical Analysis

The statistical analysis was utilized by using one-way ANOVA in the Minitab software ver. 16.2.3 (^©^2012 Minitab Inc.; Philadelphia, PA, USA). Tukey’s test was used to evaluate the significant differences (*p* < 0.05) among the test sample. The results were expressed as mean values and standard deviations.

## 5. Conclusions

This study observed that *C. hindsii* is an abundant source of α-amyrin and β-amyrin. The use of column chromatography, GC-MS, ESI-MS, and NMR optimized the effectiveness of extraction and identification this mixture. Especially, the use of NMR for quantification was impressing to determine the high content of α-amyrin and β-amyrin in *C. hindsii.* Moreover, the anti-xanthine oxidase and anti-tyrosinase potentials of α-amyrin and β-amyrin were reported for the first time and could contribute to the development of novel drugs for preventing gout and skin hyperpigmentation.

## Figures and Tables

**Table 1:** α-Amyrin and β-amyrin identified by GC-MS.

Time (min)	Peak Area (% of Total)	Compounds	Formula	Molecular Weight (g/mol)	Chemical Class
28.92	40.61	β-Amyrin	C_30_H_50_O	426.70	Triterpene
29.53	59.10	α-Amyrin	C_30_H_50_O	426.70	Triterpene

**Table 2:** ^13^C-NMR data for α-amyrin and β-amyrin in CDCl_3_.

Position	β-Amyrin	α-Amyrin	Position	β-Amyrin	α-Amyrin
1	38.60	38.80	16	26.20	26.60
2	27.20	27.30	17	32.70	33.80
3	79.00	79.10	18	47.20	59.10
4	39.80	38.80	19	46.80	39.70
5	55.20	55.20	20	31.10	39.60
6	18.40	18.40	21	34.70	31.30
7	32.50	32.90	22	37.10	40.00
8	41.70	40.00	23	28.10	28.10
9	47.60	47.70	24	15.60	15.70
10	37.00	36.90	25	15.50	15.60
11	23.70	23.30	26	16.80	17.40
12	121.70	124.40	27	26.00	23.40
13	145.20	139.60	28	28.40	28.80
14	42.80	41.50	29	33.30	16.90
15	26.90	28.10	30	23.50	21.40

**Table 3:** Quantification of α-amyrin and β-amyrin in *C. hindsii* leaves.

Content of Mixture (g/kg Dry Weight)	Ratio (%)		Contents (g/kg Dry Weight)	
10.75	α-Amyrin	β-Amyrin	α-Amyrin	β-Amyrin
	69.60	30.40	7.48	3.27

**Table 4:** Antioxidant, XO, and tyrosinase inhibitory capacities of α-amyrin and β-amyrin isolated from *C. hindsii*.

Sample	Antioxidant Activity		XO IC_50_ (µg/mL)	Tyrosinase IC_50_ (µg/mL)
	DPPH IC_50_ (µg/mL)	ABTS IC_50_ (µg/mL)		
α-Amyrin and β-Amyrin	125.55 ± 0.98 ^a^	155.28 ± 1.01 ^a^	258.22 ± 2.28 ^a^	178.85 ± 3.28 ^a^
Allopurinol		-	7.58 ± 1.29 ^b^	
Kojic acid	-	-		15.55 ± 2.28 ^b^
BHT	8.22 ± 0.89 ^b^	53.40 ± 1.52 ^b^	-	-

Values represent means ± SD (standard deviation). Values with similar superscript letter in each column are not significantly different (*p* < 0.05) (n = 3). -: measurement was not conducted; BHT: butylated hydroxytoluene; DPPH: 2,2-diphenyl-1-picrylhydrazyl; ABTS: 2,20-azinobis (3-ethylbenzothiazoline-6-sulfonic acid; and XO: xanthine oxidase.

## Data Availability

All data are presented in the article.

## Sample Availability

Samples of the compounds are available from the authors. Samples was obtained through the process of conducting experiments.
